# Data on mitochondrial respiratory function in skeletal muscle of adult male mice in response to 3-weeks heat stress

**DOI:** 10.1016/j.dib.2024.110091

**Published:** 2024-01-24

**Authors:** Rikuhide Koma, Tatsuki Matsumoto, Ayaka Yamazaki, Tsubasa Shibaguchi, Thomas Jue, Kazumi Masuda

**Affiliations:** a Graduate School of Natural Science and Technology, Kanazawa University, Ishikawa, 920-1192, Japan; b Research Fellowship for Young Scientists, Japan Society for the Promotion of Science, Tokyo, 102-0083, Japan; c School of Teacher Education, Kanazawa University, Ishikawa, 920-1192, Japan; d Graduate School of Frontier Science Initiative, Kanazawa University, Ishikawa, 920-1192, Japan; e Institute of Liberal Arts and Science, Kanazawa University, Ishikawa, 920-1192, Japan; f Department of Biochemistry and Molecular Medicine, University of California Davis, Davis, 95616-8635, USA; g Institute of Human and Social Sciences, Kanazawa University, Ishikawa, 920-1192, Japan

**Keywords:** Isolated mitochondria, Oxygraph-2k, Triceps surae, Mitochondrial oxygen consumption

## Abstract

Chronic heat stress induces mitochondrial adaptation in skeletal muscle. However, the effect of chronic heat stress on the respiratory function per mitochondria in skeletal muscle has not been well studied. Here, the present study reports on the effect of 3-weeks heat stress on muscle mitochondrial respiration using male C57BL/6JJ mice at age 21 weeks. Mice were randomly assigned to either the control group (n = 6) or passive heat group (n = 6). After 3-weeks of heat stress, the right triceps surae was removed and used for biochemical analysis. Protein expression was assessed by immunoblotting. Mitochondrial respiratory function was measured by Oxygraph-2k. The study also shows the impact of the heat stress on daily feeding, body weight, muscle weight, and protein expression of heat shock proteins (heat-response marker).

Specifications Table

**Table:** 

Subject	Biology
Specific subject area	Skeletal muscle biology
Data format	Analysis data
Type of data	Graphs, Figures and Table
Data collection	Immunoblotting, Oxygraph-2k (Oroboros, Innsbruck, Austria)
Data source location	Kanazawa, Ishikawa, Japan
Data accessibility	Repository name: Zenodo Data identification number: 10.5281/zenodo.10361238 Direct URL to data: https://zenodo.org/records/10361238

## 1 Value of the Data

• The data firstly show that passive heat stress increased the respiratory function per mitochondria in skeletal muscle.
• The data contribute to the development of heat therapy aimed at improving mitochondrial function in skeletal muscle.
• Researchers and healthcare workers may benefit from the dataset in the establishing conditions for heat therapy.
• The data also provide a protocol for measuring the respiratory function of isolated mitochondria from mouse skeletal muscle.

## 2 Data Description

**Fig. 1:**
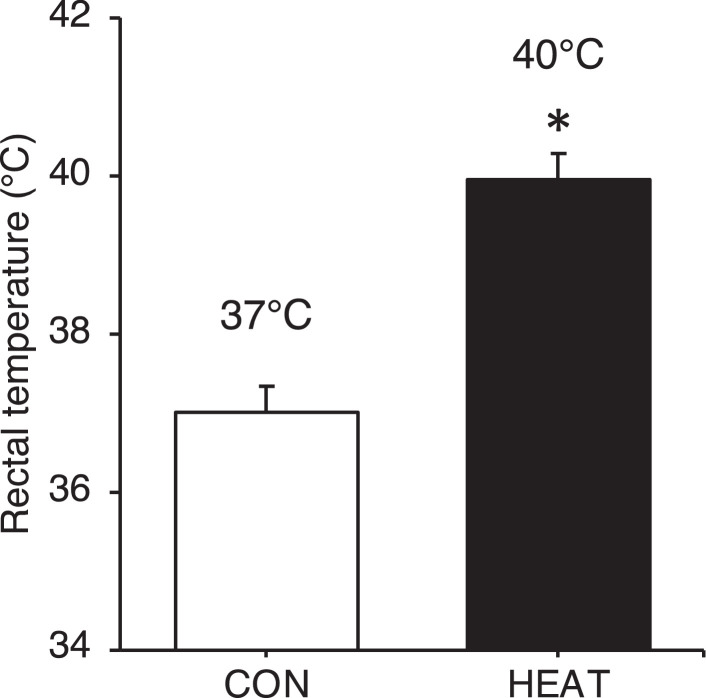
Single heat stress increases rectal temperature. Rectal temperature after the first treatments was significantly higher in the HEAT group than the CON group. The raw data for Fig. 1 are available in Excel file “Raw data (Fig. 1-4)” uploaded to Zenodo (https://zenodo.org/records/10361238). Values are presented as mean ± standard deviation (n = 6 in each group). * indicates significant difference from the CON group (p < 0.05). CON: control, HEAT: heat treatment.

**Fig. 2:**
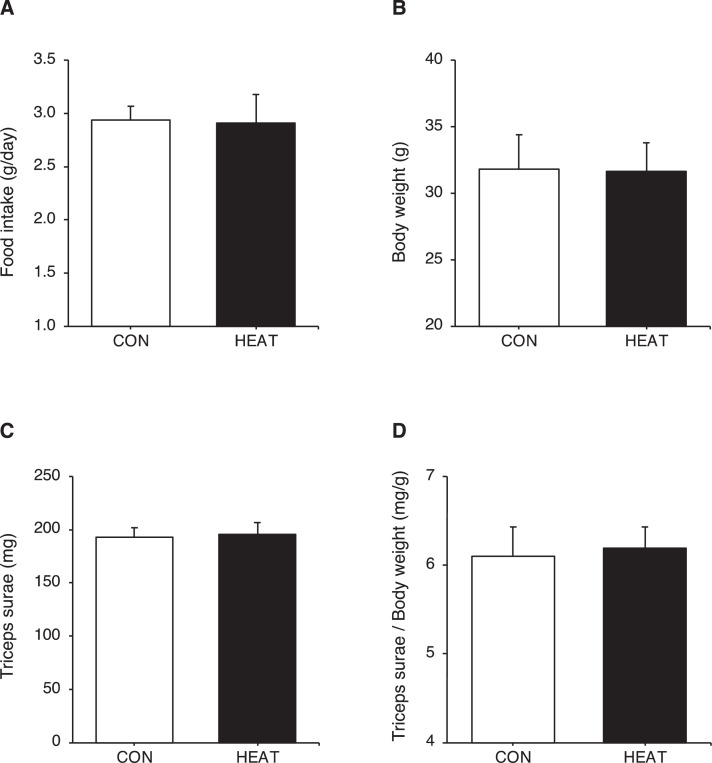
Effect of 3-weeks heat stress on food intake, body weight and triceps surae wet weight. Three-weeks heat stress did not change food intake (A), body weight (B), triceps surae wet weight, (C) and triceps surae wet weight/body weight (D). The raw data for Fig. 2 are available in Excel file “Raw data (Fig. 1-4)” uploaded to Zenodo (https://zenodo.org/records/10361238). Values are presented as mean ± standard deviation (n = 6 in each group). CON: control, HEAT: heat treatment.

**Fig. 3:**
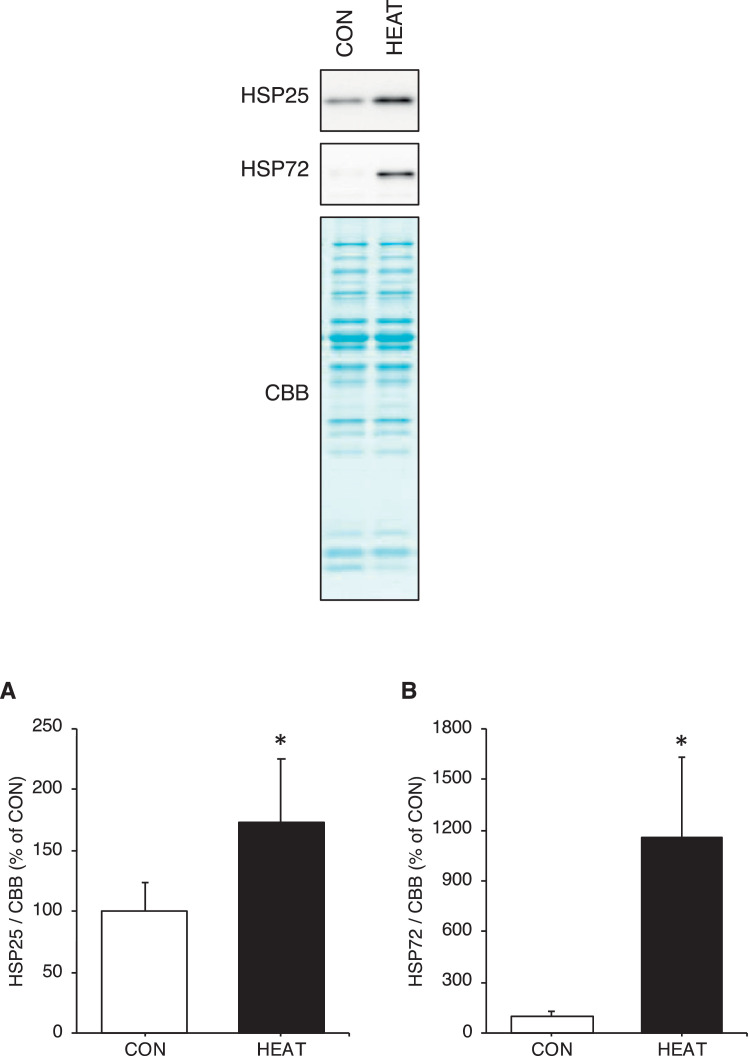
Three-weeks heat stress increases cytosolic HSPs expression in the cytosolic fraction. Expressions of cytosolic HSPs, HSP25 (A) and HSP72 (B), were significantly higher in the HEAT group than the CON group. All calculated data were normalized to CBB staining. The raw data for Fig. 3 are available in Excel file “Raw data (Fig. 1-4)” uploaded to Zenodo (https://zenodo.org/records/10361238). Unprocessed blot images are also available in Power Point file “Uncropped blot images” uploaded to Zenodo (https://zenodo.org/records/10361238). The mean value in CON group was set as 100%. Values are presented as mean ± standard deviation (n = 6 in each group). * indicates significant difference from the CON group (p < 0.05). CBB: coomassie brilliant blue, CON: control, HEAT: heat treatment, HSP: heat shock protein.

**Fig. 4:**
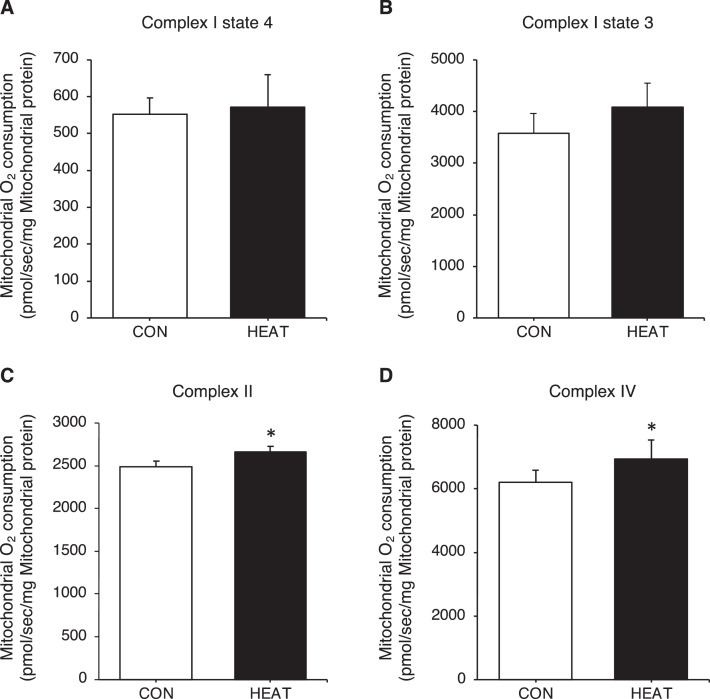
Three-weeks heat stress increases complex II- and complex IV-dependent O_2_ consumption of the isolated mitochondria. The complex I-dependent O_2_ consumption of the isolated mitochondria in both states 4 (A) and 3 (B) were similar between the CON and HEAT groups. The complex II- (C) and complex IV-dependent O_2_ consumption (D) were significantly higher in the HEAT group compared to that in the CON group. The raw data for Fig. 4 are available in Excel file “Raw data (Fig. 1-4)” and “Raw data (Oxygraph-2k)” uploaded to Zenodo (https://zenodo.org/records/10361238). Values are presented as mean ± standard deviation (n = 6 in each group). * indicates significant difference from the CON group (p < 0.05). CON: control, HEAT: heat treatment, O_2_: oxygen.

The data in Fig. 1 show the change in rectal temperature in response to single heat stress. Rectal temperature immediately after the first treatment in the heat treatment (HEAT) group is significantly higher than that in the control (CON) group (Fig. 1; 40.0°C versus 37.0°C, p < 0.05). The data in Fig. 2 show the changes in food intake, body weight, and muscle weight in response to 3-weeks heat stress. No significant differences are observed in food intake, body and muscle weight between the CON and HEAT groups (Fig. 2A-D). The data in Fig. 3 show the change in protein expression of cytosolic heat shock proteins (HSPs) in response to 3-weeks heat stress. Protein expression of heat-response markers, HSP25 and HSP72, is significantly higher in the HEAT group than in the CON group (Fig. 3A-B; p < 0.05). The data in Fig. 4 show the change in respiratory function of isolated mitochondria in response to 3-weeks heat stress. The complex I-dependent oxygen (O_2_) consumption in both states 4 and 3 are similar between the CON and HEAT groups (Fig. 4A-B). In contrast, the complex II- and complex IV-dependent O_2_ consumption are significantly higher in the HEAT group compared to that in the CON group (Fig. 4C-D; p < 0.05). The raw data for Fig. 1-4 are available in Excel file “Raw data (Fig. 1-4)” and “Raw data (Oxygraph-2k)” uploaded to Zenodo (https://zenodo.org/records/10361238). Unprocessed blot images in Fig. 3 are also available in Power Point file “Uncropped blot images” uploaded to Zenodo (https://zenodo.org/records/10361238).

## 3 Experimental Design, Materials and Methods

### 3.1 Animals

All experimental procedures and animal care were approved by the Ethics Committee on Animal Experimentation of Kanazawa University (Protocol: AP-234440). Male C57BL6/JJ mice, aged 5 weeks, were purchased from Sankyo Labo Service (Toyama, Japan). All mice were housed in cages (Depth: 205 mm, Width: 93 mm, Height: 127 mm; TM-PC-S; Tokiwa, Tokyo, Japan) of 1 mouse/cage with automatically controlled temperature (23 ± 2°C), humidity (50 ± 5%) and 12-hour light/dark cycle (lights on at 5.00 am). They were placed with free access to food (MF; Oriental Yeast, Tokyo, Japan) and water. After 16 weeks of housing mice (21 weeks) were randomly assigned to either the CON group (n = 6) or HEAT group (n = 6).

### 3.2 Experimental procedure

**Fig. 5:**
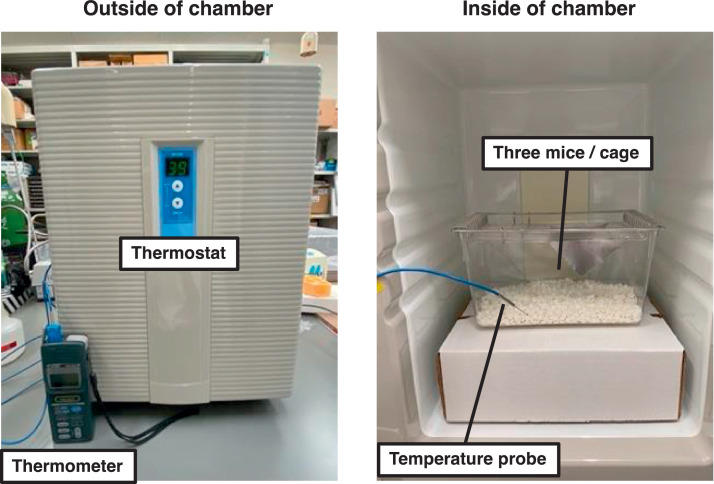
Details of thermal control chamber. All the treatments in the present study were performed using the thermal control chamber. Mice in the CON group in the cage (3 mice/cage) were placed in a normal environmental chamber (24.0 ± 0.5°C). Mice in the HEAT group in the cage (3 mice/cage) were placed in a hot environmental chamber (39.0 ± 1.0°C). CON: control, HEAT: heat treatment.

After the grouping, heat stress treatment was performed for 3 weeks (5 days/week). Mice in each group were moved to other cages (Depth: 208 mm, Width: 136 mm, Height: 115 mm; CL-0113-2; CLEA, Tokyo, Japan; 3 mice/cage) before the treatments. Mice in the HEAT group in the cage were placed in a hot environmental chamber (39.0 ± 1.0°C) for 30 min/day without anesthesia. Mice in the CON group in the cage were placed in a normal environmental chamber (24.0 ± 0.5°C) for 30 min/day. During these treatments, mice had no access to food and water. Details of the thermal control chamber (CN-25C; Mitsubishi Electric Engineering, Tokyo, Japan) are shown in Fig. 5. After these treatments, mice were returned to their original housing cage. We measured rectal temperature of mice using Rectal Probe for Mice (RET3; Physitemp, Clifton, NJ, USA) connected to Digital Thermometer (Yokogawa Test & Measurement Corporation, Tokyo, Japan) immediately after the first treatments. The food consumption of each group was also measured daily during the experimental period.

### 3.3 Cytosolic and mitochondrial preparation

Forty hours after the last heat stress treatment, mice were weighed and subsequently anesthetized with 2-5% isoflurane (VIATRIS, Tokyo, Japan). Under anesthesia, right triceps surae muscles (soleus, plantaris, gastrocnemius) were collected from both CON and HEAT groups. The muscles were washed in ice-cold saline and cleaned. Excess fat, connective tissues, and nerves were removed. Muscle wet weight was then measured. This process took less than 5 minutes. Subsequently, cytosolic and mitochondrial preparation was performed based on our previous study [1]. The muscles were minced and homogenized in 19 volumes of ice-cold Solution A (250 mM sucrose, 5 mM NaN_3_, 2 mM EGTA, 20 mM HEPES-Na, pH 7.4) with twenty strokes of a Teflon pestle in a Potter-Elvehjem glass tissue homogenizer at 1,000 rpm. The homogenate was centrifuged at 600 *g* for 10 min at 4°C to remove nuclei and debris. The supernatant was further centrifuged at 16,000 *g* for 30 min at 4°C. The resultant supernatant was collected and used as cytosolic fraction in immunoblotting analysis. The pellet was washed twice in Solution A, re-suspended in Solution A and then re-centrifuged at 16,000 *g* for 30 min at 4°C to precipitate mitochondria. The pellet was washed twice in Solution A and re-suspended in Solution A. The crude suspension was immediately used for mitochondrial respiration analysis.

### 3.4 Mitochondrial respiration analysis

Mitochondrial respiratory capacity was measured using a Oxygraph-2k (Oroboros, Innsbruck, Austria) at 37°C according to the modified method of previous studies [2]. Just prior to the assay, the protein concentration of the crude mitochondrial suspension was determined by the Bradford, protein assay kit (Bio-Rad Laboratories, Tokyo, Japan). The crude suspension was adjusted to a final concentration of 1 mg/mL mitochondrial protein with Solution A. The mitochondrial suspension was added to the Oxygraph chambers containing MiR05 solution (0.5 mM EGTA, 3 mM MgCl_2_, 60 mM lactobionic acid, 20 mM taurine, 10 mM KH_2_PO_4_, 20 mM HEPES, 110 mM D–sucrose, 1 g/L bovine serum albumin, pH 7.1) to a final concentration of 0.025 mg/mL. The chambers were calibrated daily with MiR05 solution. The solution was air saturated with O_2_ concentrations of approximately 190 µM before the start of the experiment. Malate (final concentration: 5 mM) and glutamate (final concentration: 10 mM) were then added to induce state 4 respiration, which is dependent on the complex I. Next, ADP (final concentration: 1 mM) was added to the chambers to induce state 3 respiration. After inhibition with rotenone (final concentration: 0.2 µM), succinate (final concentration: 10 mM) was added to activate respiration via complex II. Subsequently, antimycin A (final concentration: 2.5 µM) was added to the chambers to inhibit complex III, which then blocks complex I to III-dependent respiration. Complex IV-dependent O_2_ consumption was monitored after the addition of ascorbate (final concentration: 1 mM) and TMPD (final concentration: 250 µM). TMPD was added after addition of ascorbate to the chamber to provide electrons to cytochrome c. The ascorbate inhibited additional oxidation. O_2_ consumption rates were normalized to 50 µg of mitochondrial protein within the chamber to evaluate changes in respiratory function per mitochondria. In the experiments shown, respiratory control ratio (state 3/state 4) of all samples was above 4 (Table 1), indicating evidence of a viable mitochondrial preparation [3].

**Table 1:** Respiratory control ratio (state3/state4) of all samples.

Sample	CON	HEAT
1	6.7	8.5
2	7.0	6.3
3	7.7	9.5
4	5.6	5.5
5	7.5	8.8
6	4.9	5.6
Average	6.6	7.4
SD	1.1	1.8

CON: control, HEAT: heat treatment, SD: standard deviation

### 3.5 Immunoblotting

Cytosol samples were solubilized in 2x SDS-PAGE loading buffer [125 mM Tris, 4% (w/v) SDS, 20% (w/v) glycerol, 10% (v/v) β-mercaptoethanol, 0.002% (w/v) bromophenol blue, pH 6.8] and incubated for 5 min at 95°C before subjecting to immunoblotting analysis. Equal amounts of proteins (2 µg/lane) were separated by 13% SDS-PAGE, and then the separated proteins were electrophoretically transferred onto polyvinylidene difluoride membranes (Clear Blot Membrane-P plus; ATTO, Tokyo, Japan) using a semi-dry system (WSE-4045 HorizeBLOT 4M; ATTO). To check for equal protein loading, the membranes were stained with Coomassie brilliant blue (CBB) using EzStain AQua MEM (ATTO) according to the standard manufacturer procedure. The stained membrane was completely dried and digital images were obtained with a scanner (GT-S650, Seiko Epson, Nagano, Japan). After re-hydrating and de-staining the membranes by 100% methanol, the membranes were washed with Tris-buffered saline (150 mM NaCl, 25 mM Tris-HCl, pH 7.4) containing 0.1% (v/v) Tween-20 (TBS-T) for 5 min and blocked with Bullet Blocking One (Nacalai Tesque, Kyoto, Japan) for 5 min at room temperature. After the blocking, the membranes were washed twice with TBS-T for 5 min, and then incubated with rabbit polyclonal antibodies against HSP25 (1:1,000; AD1-SPA-801; Enzo Life Sciences, Farmingdale, NY, USA) and HSP72 (1:2,000; 10995-1-AP; Proteintech, Tokyo, Japan) for overnight at 4°C. These antibodies were diluted in TBS-T containing 5% (w/v) bovine serum albumin and 0.02% (w/v) NaN_3_. After the overnight incubation, the membranes were washed with TBS-T for 5 min twice and reacted with horseradish peroxidase-conjugated anti-rabbit IgG (1:5,000; #7074; Cell Signaling Technology, Beverly, MA, USA) in TBS-T containing 5% (w/v) skim milk for 1-h at room temperature. Following three washes with TBS-T for 7 min, protein signals were visualized by the chemiluminescence detection method using the WSE-7120 EzWestLumi plus (ATTO) and then captured with MicroChemi (Berthold Technologies, Bad Wildbad, Germany). Immunoreactivities and CBB staining intensities were quantified using Image J software (version 1.53a, National Institutes of Health, Bethesda, MD, USA). The band intensity of each protein was normalized by CBB staining intensity. The mean value in CON group was set as 100%.

### 3.6 Statistical analysis

All data are presented as the mean ± standard deviation. All statistical analyses were performed using IBM SPSS Statistics software (Advanced Analytics Inc., Tokyo, Japan). The normality of the data was tested using the Shapiro-Wilk normality test. If the Shapiro-Wilk normality test passed, the statistical differences were tested by unpaired t-test. If not, the nonparametric Mann-Whitney U test was used. The significance was set at p < 0.05.

## Limitations

The present study used only male mice and it is uncertain whether the same results would be obtained in females. Therefore, further studies are necessary to clarify the effect of chronic heat stress on skeletal muscle mitochondrial respiration in females.

## Ethics Statement

All experimental procedures and animal care were approved by the Ethics Committee on Animal Experimentation of Kanazawa University (Protocol: AP-234440). The experiments complied with the ARRIVE guidelines and were performed in accordance with the National Institutes of Health guide for the care and use of laboratory animals (NIH Publications No. 8023, revised 1978).

## CRediT authorship contribution statement

**Rikuhide Koma:** Conceptualization, Methodology, Validation, Formal analysis, Investigation, Data curation, Writing – original draft, Funding acquisition. **Tatsuki Matsumoto:** Investigation, Data curation. **Ayaka Yamazaki:** Data curation, Writing – review & editing. **Tsubasa Shibaguchi:** Writing – review & editing. **Thomas Jue:** Writing – review & editing. **Kazumi Masuda:** Supervision, Writing – review & editing, Funding acquisition.

## Data Availability

Data on mitochondrial respiratory function in skeletal muscle of adult male mice in response to 3-weeks heat stress (Original data) (Zenodo)
